# Heat shock protein 60: an endogenous inducer of dopaminergic cell death in Parkinson disease

**DOI:** 10.1186/1742-2094-11-86

**Published:** 2014-05-08

**Authors:** Carmen Noelker, Lydie Morel, Anke Osterloh, Daniel Alvarez-Fischer, Thomas Lescot, Minka Breloer, Maike Gold, Wolfgang H Oertel, Carmen Henze, Patrick P Michel, Richard C Dodel, Lixia Lu, Etienne C Hirsch, Stéphane Hunot, Andreas Hartmann

**Affiliations:** 1 CR-ICM, INSERM UMR_S1127, Université Pierre et Marie Curie Paris 06 UMR_S1127, CNRS UMR 7225, Groupe Hospitalier Pitié-Salpêtrière, 75013 Paris, France; 2 Department of Neurology, Philipps-University Marburg, 35043 Marburg, Germany; 3 Bernhard Nocht Institute for Tropical Medicine, Hamburg 20324, Germany; 4 Institute of Neurogenetics, University of Lübeck, 23562 Lübeck, Germany; 5 Department of Psychiatry, University of Lübeck, 23538 Lübeck, Germany

**Keywords:** PD, Neuroinflammation, Hsp60, Neurodegeneration, Microglia, Innate immunity

## Abstract

**Background:**

Increasing evidence suggests that inflammation associated with microglial cell activation in the substantia nigra (SN) of patients with Parkinson disease (PD) is not only a consequence of neuronal degeneration, but may actively sustain dopaminergic (DA) cell loss over time. We aimed to study whether the intracellular chaperone heat shock protein 60 (Hsp60) could serve as a signal of CNS injury for activation of microglial cells.

**Methods:**

Hsp60 mRNA expression in the mesencephalon and the striatum of C57/BL6 mice treated with MPTP (1-methyl-4-phenyl-1,2,3,6-tetrahydropyridine) and the Hsp60/TH mRNA ratios in the SN of PD patients and aged-matched subjects were measured. To further investigate a possible link between the neuronal Hsp60 response and PD-related cellular stress, Hsp60 immunoblot analysis and quantification in cell lysates from SH-SY5Y after treatment with 100 μM MPP^+^ (1-methyl-4-phenylpyridinium) at different time points (6, 12, 24 and 48 hours) compared to control cells were performed. Additional MTT and LDH assay were used. We next addressed the question as to whether Hsp60 influences the survival of TH^+^ neurons in mesencephalic neuron-glia cultures treated either with MPP^+^ (1 μM), hHsp60 (10 μg/ml) or a combination of both. Finally, we measured IL-1β, IL-6, TNF-α and NO-release by ELISA in primary microglial cell cultures following treatment with different hHsp60 preparations. Control cultures were exposed to LPS.

**Results:**

In the mesencephalon and striatum of mice treated with MPTP and also in the SN of PD patients, we found that Hsp60 mRNA was up-regulated. MPP^+^, the active metabolite of MPTP, also caused an increased expression and release of Hsp60 in the human dopaminergic cell line SH-SY5Y. Interestingly, in addition to being toxic to DA neurons in primary mesencephalic cultures, exogenous Hsp60 aggravated the effects of MPP^+^. Yet, although we demonstrated that Hsp60 specifically binds to microglial cells, it failed to stimulate the production of pro-inflammatory cytokines or NO by these cells.

**Conclusions:**

Overall, our data suggest that Hsp60 is likely to participate in DA cell death in PD but via a mechanism unrelated to cytokine release.

## Background

Parkinson disease (PD) is a common neurodegenerative disorder clinically characterized by akinesia, rigidity and rest tremor [1]. The cardinal neuroanatomical feature of PD is a massive and preferential loss of dopaminergic (DA) neurons in the substantia nigra pars compacta (SNpc) of patients, resulting in a drastic decline in striatal dopamine concentrations.

Another histopathologic hallmark of PD is microglial activation in the SNpc [2,3]. Activated microglia are believed to contribute to the neurodegenerative process through the release of pro-inflammatory and/or cytotoxic factors such as IL-1β, TNF-α, nitric oxide (NO) and reactive oxygen intermediates [2]. Moreover, several observations suggest that an ongoing stimulus could lead to disease progression long after the initial toxic insult [4-6], thereby amplifying and sustaining neuroinflammation and, ultimately, leading to destruction of nigral DA neurons. These processes are generally considered to be a non-specific consequence of neuronal death. But the possibility that *suffering/damaged* DA neurons might also participate in the activation of microglia and in sustaining neuroinflammation deserves consideration.

In light of our knowledge about the innate immune system, it is highly plausible to assume that Toll-like receptors (TLRs) are major mediators in glial cells triggering the release of cytokines that ultimately kill DA neurons in the SNpc. Accordingly, as first demonstration in the central nervous system (CNS), the only cellular population that stained positive for Toll-like receptor 4 (TLR4) in the brain parenchyma of adult rats were microglia [7].

Traditionally, it has been considered that the danger-associated molecules sensed by TLRs are highly conserved, so called pathogen-associated molecular patterns (PAMPs), which are expressed by bacteria, viruses, or other pathogens but are not present in mammalian cells [8]. For example, bacterial lipopeptides (BLPs), lipopolysaccharide (LPS), and flagellins are recognized by TLR2, TLR4, and TLR5, respectively. A number of reports have emerged to suggest that diverse molecules of host-cell origin may also serve as endogenous ligands of TLR2 or TLR4 [9]. To date, there have been at least 23 reports of distinct endogenous ligands of TLR2 or TLR4, representing molecules of diverse source and structure, ranging from those associated with cell damage and major extracellular matrix (ECM) turnover to inflammatory mediators and oxidatively modified lipids [10].

Recently, we have demonstrated that TLR4-deficient mice are less vulnerable to MPTP (1-methyl-4-phenyl-1,2,3,6-tetrahydropyridine) intoxication than wild-type mice and display a decreased number of Iba1^+^ and MHC II^+^ activated microglial cells after MPTP application, suggesting that the TLR4 pathway is involved in experimental PD. However, the TLR4 ligand responsible for this activation remains elusive [7].

Neuronal activators of the TLR4-dependent pathway in microglia could be heat shock proteins (Hsps), a group of highly conserved proteins that are constitutively expressed in most cells under physiological conditions. They are commonly induced by the presence of denatured proteins and contribute to the restoration of the tertiary structure and enzymatic activity of these proteins [11]. Upon cellular stress, Hsps are up-regulated and released from suffering/damaged cells [12,13]. Beside the assumption that the Hsp response to cell injury plays a beneficial role in cell survival, recent evidence suggests that Hsps can also exert immunomodulatory functions [14]. Hsp60 - which is typically located in mitochondria, a primary subcellular pathogenic locus in PD [15] - was found to be aberrantly expressed on the cell surface of neuronal cells in response to stress [16,17]. Interestingly, Hsp60 binds to microglia and the microglial receptor for Hsp60 has been suggested to be TLR4 [18]. In addition, a recent study demonstrated that Hsp60 serves as an endogenous signal of injury in the CNS by activating microglia via a TLR4- and myeloid differentiation factor 88 (MyD88)-dependent pathway [19]. This mechanism may account for the triggering of inflammatory responses in the vicinity of suffering/damaged DA neurons and engage a neuronal-glial crosstalk. We therefore investigated the role of Hsp60 in this scenario which may be released or expressed at the cell surface of suffering/damaged DA neurons and contribute to their demise.

## Material and methods

### Animals and MPTP treatment

Adult (eight to ten weeks) male C57/BL6 mice (Janvier Breeding Centre, France) were housed, handled, and cared for in accordance with the Guide for the Care and Use of Laboratory Animals (NCR (National Research Council) 1996) and the European Union Council Directive 86/609/EEC, and the experimental protocols were carried out in compliance with institutional ethical committee guidelines for animal research. For all studies, mice were maintained on a 12:12 hour light/dark cycle with lights on at 6.30 am. The room temperature was kept at 23°C, with free access to standard diet (LASQCdiet® Rod16-R, LASvendi) and tap water. Mice (n = 8 to 10 per group) were treated using the acute MPTP paradigm (4 × 20 mg/kg MPTP-HCl (Sigma-Aldrich, Lyon, France) every 2 hours) [20,21] and animals were sacrificed after 12, 24, 48, 96 or 168 hours after the last injection. The brain was excised and the specific brain regions dissected. The corresponding controls were collected variously across the days.

### Hsp60 mRNA-PCR in the striatum and SN of mice

Striatum and SN of the mice were dissected. Tissues were then placed into Trizol reagent (Invitrogen, Cergy Pontoise Cedex, France), homogenized, and total RNA was prepared according to the manufacturer’s instructions. First-strand cDNA was synthesized from 1 μg of total RNA using the ThermoScript RT-PCR system (Invitrogen, Cergy Pontoise Cedex, France). To access expression levels specific designed intron-spanning primers for Hsp60 (fwd = 5′-CAC AGT GAA GGA TGG AAA AAC CCT-3′ and rev = 5′-TCT TTG GTG ACA ATG ACC TCC C-3′) and tubulin (fwd = 5′-TGT CCA TGA AGG AGG TGG ATG AG-3′ and rev = 5′-ATG TTG CTC TCA GCC TCG GTG AAC-3′) were used. The annealing temperature was 64°C and the number of PCR cycles was chosen to stop the reaction in the linear phase of amplification (25 cycles). The amplification of Hsp60 and tubulin was done in the same reaction for each sample. After gel electrophoresis, digital images were analyzed with NIH ImageJ software for quantification.

### Hsp60 qRT-PCR of human SN

The Hsp60 and TH primers for quantitative PCR were designed by primer express 3.0 (Applied Biosystems, Darmstadt, Germany) and their specificity was confirmed by blastn analysis. Their sequences were as followed: Hsp60_fwd: 5′-GCC GCC CCG CAG AA-3′, Hsp60_rev: 5′-CCT GGA CAC CGG TCT CAT CT-3′, TH_fwd: 5′-GCA CCT TCG CGC AGT TCT-3′, TH_rev: 5′-ACA GCG TGG ACA GCT TCT CA-3′. Mesencephalon ss PD patients and age-matched control subjects (n = 5 per group) were provided by the INSERM UMR_S1127 brain bank. The SN was dissected from frozen slides. Total RNA was extracted with Trizol (Invitrogen, Cergy Pontoise Cedex, France) and the quantity and quality were determined by Nanodrop and Agilent, respectively. One microgram of total RNA was reverse-transcribed with Superscript III RT-kit (Invitrogen, Cergy Pontoise Cedex, France) according to the manufacturer’s instructions. qPCR was performed with SYBR GreenER TM qPCR SuperMix kit (Invitrogen, Cergy Pontoise Cedex, France) in ABI 7500 real time thermal cycler (Applied Biosystems, Darmstadt, Germany). The relative mRNA expression levels were normalized by the geometric mean of two housekeeping genes (GAPDH and HPRT) with qBaseplus software (Primerdesign, Southampton, UK).

### SH-SY5Y cell cultures

SH-SY5Y (p3-p7) cells [22] were maintained in DMEM (Gibco, Saint Aubin, France) supplemented with 15% FCS, 1 mM L-glutamine, 100 U of penicillin/ml, and 100 μg of streptomycin sulfate/ml and equilibrated with 5% CO_2_-95% air at 37°C. At different time points, the cells were treated with 100 μM MPP^+^ (1-methyl-4-phenylpyridinium) [23] purchased from Sigma-Aldrich (Lyon, France).

### Primary mesencephalic cell cultures

Mesencephalic cultures were prepared from the ventral midbrain of Swiss mouse embryos at gestational age 13.5 days (Janvier Breeding Center, France). The dissected tissue pieces were processed according to previously described protocols [24,25]. Briefly, after mechanical dissociation in modified L15 medium [26] with no enzymatic treatment, the cells in suspension were plated at a density of 1.5 to 2.0 × 10^5^ cells/cm^2^ in polyethylenimine (PEI, Sigma-Aldrich, Lyon, France; 1 mg/mL) precoated culture plates (24 well-plates). The cells were then allowed to mature and differentiate in N5 culture medium [25,27], supplemented with 5% horse serum and 0.5% fetal calf serum (FCS, endotoxin level of the used FCS < 10 EU/ml) except for the first three days *in vitro* (DIV), when the concentration of FCS was raised to 2.5% to favor cell attachment and initial development. Mesencephalic DA neurons degenerate spontaneously and progressively when maintained in N5 culture medium supplemented with serum proteins, whereas other types of neurons survive. The death of these neurons occurs spontaneously through a mechanism that is dependent on glial cells [24,28]. It could be shown previously that depolarization by elevation of extracellular K^+^ ([K^+^] 30 mM) was efficient in preventing DA cell demise. Note that K^+^-induced depolarization was performed in the presence of 1 μM MK-801 to prevent secondary excitotoxic stress. In some experiments, Ara-C (2 μM) was added to the medium at DIV 1 to 2 after plating to inhibit proliferation of non-neuronal cells (astrocytes, microglia) as well in the presence of 1 μM MK-801 to prevent secondary excitotoxic stress [28].

Mesencephalic cultures were treated either with 1 μM MPP^+^ at DIV 4 and DIV 5 as previously described [29], 10 μg/ml low-endotoxin recombinant human Hsp60 dissolved in PBS (hHsp60; Loke Diagnostics ApS, Risskov, Denmark) at DIV 2 to DIV 5 or in combination of both. The hHsp60 contained < 2 endotoxic units of LPS/mg of protein as determined by limulus amoebocyte lysate assay (BioWhittaker, Cologne, Germany). Since hHsp60 has a much weaker effect than MPP^+^ on cell death, we decide to prolonged treatment of the cell cultures compared to MPP^+^ treatment. At DIV 5, medium including MPP^+^ and hHsp60 was removed and cultures were left to recover until DIV 10 in control medium. Trypsin proteolysis (Gibco, Saint Aubin, France) or pre-treatment by boiling of hHsp60 were used to control for Hsp60-mediated effects, as well as additional treatment with polymyxin B (10 μg/ml, PMBS, Sigma-Aldrich, Lyon, France) as a specific LPS inhibitor to control for LPS effects.

### Highly enriched microglial cultures of mice ventral mesencephalon

Almost pure microglial cultures were obtained using a technique of high-yield isolation of microglia by mild trypsinization [30]. Briefly, neuronal/glial mesencephalic cultures were prepared as described previously except for the culture medium, which was DMEM/F12 nutrient mixture (DMEM/F12; Invitrogen, Cergy Pontoise, France) supplemented with 10% FCS (endotoxin level of the used FCS < 10 EU/ml). After DIV 14, the cultures were washed for 1 minute with DMEM/F12 to eliminate serum and then incubated with a trypsin/EDTA solution (0.25% trypsin, 1 mm EDTA in HBSS; Invitrogen, Cergy Pontoise, France) diluted 1:4 in DMEM/F12 for half to one hour at 37°C until the upper layer (mainly constituted by neurons/astrocytes) was detached. The medium containing the layer of detached cells was aspirated and the highly enriched microglial cell population (98% of pure microglial cells) that remained attached to the bottom of the well was exposed to 500 μl of DMEM/F12 with 10% FCS to allow trypsin inactivation. The cells were treated with low-endotoxin recombinant human Hsp60 (hHsp60) 10 μg/ml and contained < 2 endotoxic units of LPS/mg of protein as determined by limulus amoebocyte lysate assay (BioWhittaker, Cologne, Germany) or LPS 0.1 μg/ml (Sigma-Aldrich, Lyon, France, strain 055:B5), both dissolved in PBS.

### MTT and LDH assays

MTT (3-[4,5-dimethylthiazol-2-yl]-2,5-diphenyl-tetrazolium bromide, Sigma-Aldrich, Lyon, France) was added to the cells at a final concentration of 0.25 mg/ml and incubated for 1 hour [31-33]. Thereafter, the cells were lysed and quantified using a microplate reader (MultiskanReader, ThermoLabsystems, Egelsbach, Germany) at a wavelength of 570 nm. Cell death and cell lysis was based on lactate dehydrogenase (LDH) activity released into the supernatant. LDH activity was measured using the CytoTox 96® Non-Radioactive Cytotoxicity Assay (Promega, Lyon, France) according to the manufacturer’s protocol.

### Western blots

For Western blotting, cellular protein was isolated using the lysis buffer M-PER Mammalian Protein Extraction Reagent according to the manual (Pierce, Perbio, Brebières, France). Twenty micrograms total protein/lane was loaded onto a reducing 12% SDS-PAGE and electroblotted onto nitrocellulose membranes. After blotting, the internal protein loading controls were prepared according to the manual of MEM Code, Pierce (Perbio, Brebières, France). Afterwards, the membranes were blocked and incubated for 24 hours at 4˚C with a monoclonal mouse anti-Hsp60 antibody (0.5 μg/ml; R&D Systems,Wiesbaden, Germany) and for the loading control with an anti-beta-actin polyclonal rabbit antibody (1:2,000, Sigma-Aldrich, Lyon, France), respectively. For detection, the nitrocellulose membranes were washed and incubated with a HRP-conjugated secondary antibody (Jackson ImmunoResearch, West Grove, PA, USA; goat-anti-mouse or goat-anti-rabbit both 1:50,000). After AB-incubation membranes were incubated in Super Signal Ultra substrate working solution (Perbio, Brebières, France) and exposed to an autoradiographic film (Mat Plus DG Film, Kodak, Maisons-Alfort, France). Afterwards, Western blot signals were analyzed by densitometry using the NIH Image software Scion, and the Hsp60/α-actin ratio was calculated.

### Immunocytochemical procedures *in vitro*

All cell cultures were fixed for 15 minutes at room temperature with formaldehyde (4% in PBS) and washed three times with PBS. For primary mesencephalic cell cultures, the survival of DA neurons was determined by tyrosine hydroxylase (TH) immunocytochemistry. The cultures exposed first for 24 hours at 4°C to a rabbit-polyclonal anti-TH antibody (Pel-Freez Biologicals, Rogers, AR, USA) diluted 1:1,000 in PBS containing 0.2% Triton X-100, were then incubated for one hour at room temperature with an anti-rabbit IgG Alexa488 conjugate (1:500; Sigma-Aldrich/RBI, Lyon, France). In order to identify the total number of neurons in the cultures, cultures were incubated for 48 hours at 4°C with a mouse anti-neuronal Nuclei (NeuN) biotin conjugated monoclonal antibody (Chemicon International, Limburg, Germany) and then incubated with a streptavidin-Cy3-antibody (1:1,000, Jackson ImmunoResearch, West Grove, PA, USA) for 1 hour at room temperature. Mesencephalic cultures contained between 1 and 3% TH^+^ cells at the time of plating. To quantify the cells, the entire TH^+^ and/or NeuN^+^ neurons of the used wells were counted.

In the primary microglial cultures, microglial cells were identified with a rat anti-CD11b Ig (MAC-1; Serotec, Oxfordshire, UK; 1:100 in PBS, 24 hours at 4°C) and revealed using an anti-rabbit IgG Alexa488 conjugate (1:500; Sigma-Aldrich/RBI, Lyon, France). Note that Triton X-100 was not used for the detection of microglial cell surface markers. A polyclonal rabbit beta-tubulin antibody was used (Cell Signaling, Leiden, Netherlands; 1: 200 in PBS containing 0.2% Triton X-100, 24 hours at 4°C) to detect cytoskeletal structures and revealed using an anti-rabbit IgG Alexa488 conjugate (1:500; Sigma-Aldrich/RBI, Lyon, France).

For staining double-stranded DNA, we used PBS containing 10 μg/ml 4’6-diamidino-2-phenylindole (DAPI, Sigma-Aldrich, Lyon, France). Illustrations of microglial cells are presented as inverted images. For the confocal images, a Leica SP1 confocal microscope was used (Leica, Wetzlar, Germany).

### Hsp60 binding studies

For binding studies, labeled or unlabeled hHsp60 and BSA (Sigma-Aldrich, Lyon, France) were used. The labeled proteins were conjugated to Alexa 647 using the protein labeling kits from Molecular Probes, Saint Aubin, France, according to the manufacturer’s protocol. For binding of hHsp60 to primary microglia 1 × 106 cells were incubated on ice with either 10 μg/ml Alexa 647-labeled hHsp60 or BSA. To exclude an unspecific effect of labeled proteins, cells were also treated with unlabeled proteins (hHsp60 or BSA). Cells that obtained unlabeled hHsp60 were treated with 30 μl of Cohn II fraction (Sigma-Aldrich, Lyon, France) and stained with Hsp60-specific antibody (clone LK-1, 1:100 in PBS), TRITC-labeled goat anti-mouse secondary antibody (1:400 in PBS) and DAPI (1:1,000 in PBS). After staining cells were fixed in PBS/1% paraformaldehyde (PFA), centrifuged onto glass slides, and covered with anti-FADE solution (BiomedDia, Zweibruechen, Germany).

### Hsp60 quantification

The estimation of protein concentration was performed by the Bradford assay and verified by SDS-PAGE followed by silver staining. Western blot for Hsp60 quantification was made with a specific anti-Hsp60 monoclonal antibody (SPA-810, StressGen, San Diego, CA, USA).

### FACS staining

1 to 2 × 10^6^ microglial cells were washed with PBS, detached and incubated with different concentrations of hHsp60 (1 to 50 μg/ml) on ice for 30 minutes. Cells were washed with PBS and Fc receptors of cells were blocked with 30 μl of Cohn II fraction (10 mg/ml, Sigma-Aldrich, Lyon, France) prior to staining with a specific antibody against Hsp60 (SPA-810; StressGen, San Diego, CA, USA). To avoid an internalization of cell surface-bound Hsp60, the incubation of microglial cells with Hsp60 was done on ice. Cells were then washed and a phycoerythrin-labeled goat-anti mouse IgG (Jackson ImmunoResearch, West Grove, PA, USA) was added.

### Cytokine and Hsp60 quantification by ELISA

The supernatant of SH-SY5Y cell cultures was collected at different time points, centrifuged and immediately frozen at -80°C. The detection of Hsp60 was carried out by using a quantitative sandwich immunoassay (Hsp60-ELISA-Kit, StressGen, San Diego, CA, USA) according to the manufacturer’s protocol. Release of microglial cytokines (IL-1β, IL-6 and TNF-α) was measured at different time points (1, 2, 4, 6 and 24 hours) after addition of 10 μg/ml hHsp60.

In addition, microglial cells were treated with hHsp60 that was left untreated, boiled, trypsin-inactivated or pre-incubated with either a specific Hsp60-antibody (Clone 4B9; Dianova, Hamburg, Germany) or an isotypic IgG2-antibody (Santa Cruz Biotechnology Inc., Heidelberg, Germany). As a positive control for cytokine release, primary microglial cells were incubated with only LPS (0.1 μg/ml), a prototypical inflammogen known to activate microglia through TLR4 stimulation [34,35].

Cytokine release into the supernatant was measured employing the DuoSet ELISA Development System mouse IL-1β, IL-6 and TNF-α (R&D Systems, Wiesbaden, Germany). ELISAs were performed according to the manufacturer’s protocol.

### Measurement of NO

Accumulated nitrite, a stable oxidation product of NO, was measured using Griess reagents [36]. Fifty microliters of primary microglial cell supernatants were transferred to a 96-well microtiter plate and 50 μl of solution 1 (1% sulfanilamide in 5% phosphoric acid) were added. After 10 minutes incubation in the dark, 50 μl of solution 2 (0.1% naphthylethylenediamine dihydrochloride) were added and incubated for 10 more minutes in the dark. The absorbance was measured at 450 nm using a plate reader (ELISA-Reader Infinite® 200 series, Tecan, Crailsheim, Germany).

### Statistical analysis

All *in vitro* experiments were performed in at least three independent experiments, with a minimum of three wells per experimental condition. We confirm that there is no duplication of data presentation in the text. Data are expressed as the percent of corresponding control values. The normalization to control was used because of the high variance due to the different preparations of primary cell cultures. Each data point represents mean ± SEM. Multiple comparisons against a single reference group were performed by one-way analysis of variance (ANOVA) followed by a *post hoc* Dunnett’s test. When all pairwise comparisons were carried out, one-way ANOVA was followed by a *post hoc* Student-Newman-Keuls test. For the human qPCR experiments, a Spearman correlation test between Hsp60 and TH mRNA expression was calculated. The null hypothesis was rejected at an α risk of 5%.

## Results

### Hsp60 mRNA up-regulation is observed in MPTP-treated mice and in PD patients

**Figure 1:**
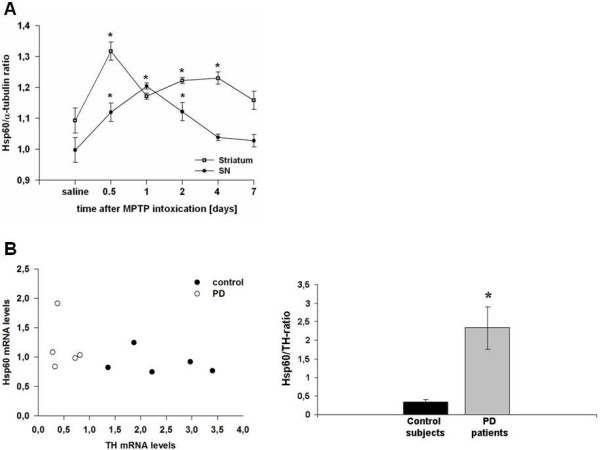
**Hsp60 mRNA expression in 1-methyl-4-phenyl-1,2,3,6-tetrahydropyridine (MPTP)-treated mice and Parkinson disease (PD) patients. (A)** Hsp60 mRNA expression in the mesencephalon (substantia nigra (SN), filled circles) and the striatum (open circles) of C57/BL6 mice treated with MPTP. Mice (n = 8 to 10 per group) were treated using the acute MPTP paradigm (4 × 20 mg/kg MPTP-HCl (Sigma-Aldrich, Lyon, France) every 2 hours) and animals were killed 12, 24, 48, 96 or 168 hours after the last injection. **(B)** Hsp60/TH mRNA ratios in the SN of PD patients and aged-matched subjects. Results were expressed as a ratio to correct for dopaminergic (DA) cell loss in PD. *P < 0.05 versus corresponding controls.

We postulated that injury of DA neurons could result in the release of Hsp60, causing activation of microglia cells. To test this hypothesis, we first examined the dynamic changes of Hsp60 mRNA expression in the mesencephalon and the striatum of C57/Bl6 mice intoxicated with MPTP. Compared to saline-treated animals, Hsp60 mRNA expression was up-regulated in the mesencephalon and striatum of MPTP-exposed mice, the striatal response being more sustained in time (Figure 1A). Thus, this suggests that MPTP-induced mitochondrial stress in nigral DA neurons can trigger an Hsp60 response *in vivo*. To confront these results with the human pathology, we further assessed the expression level of Hsp60 transcripts in human *post-mortem* SN specimen by qRT-PCR. We found a significant increase in the Hsp60/TH ratio (to correct for DA cell loss in PD) in PD patients (n = 5) compared to age-matched control subjects (n = 5) (Figure 1B) suggesting that the Hsp60 stress response may play a role in the pathomechanism of PD.

### Mitochondrial stress induces up-regulation and release of Hsp60 in the human dopaminergic cell line SH-SY5Y

**Figure 2:**
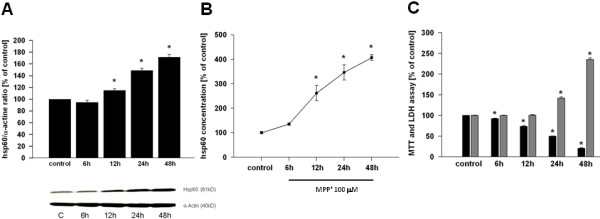
**Hsp60 induction and release after MPP**^**+ **^**treatment. (A)** Hsp60 immunoblot analysis and quantification in cell lysates from SH-SY5Y after treatment with 100 μM MPP^+^ (at 6, 12, 24 and 48 hours) compared to control cells. **(B)** Hsp60 released by cultured SH-SY5Y cells treated by MPP^+^ (100 μM, at 6, 12, 24 and 48 hours) compared to control cultures. **(C)** MTT and lactate dehydrogenase (LDH) assay in SH-SY5Y cells exposed to MPP^+^ (100 μM, at 6, 12, 24 and 48 hours) compared to control cells. MTT assay (black bars), LDH assay (grey bars). *P < 0.05 versus corresponding controls.

To further investigate a possible link between the neuronal Hsp60 response and PD-related cellular stress, we treated the human DA cell line SH-SY5Y with the mitochondrial toxin MPP^+^ and first assessed Hsp60 expression. As shown in Figure 2A, exposure of DA cells to MPP^+^ induced a time-dependent up-regulation of Hsp60 expression as assayed by immunoblot analysis on whole cell lysates. Up-regulation was first noticed 12 hours after intoxication and continuously increased up to 48 hours. The level of Hsp60 was not only increased in the intracellular compartment but also in the cell culture supernatant (Figure 2B), suggesting that cellular damage induced by mitochondrial alteration can stimulate the release of stress-related factors from diseased DA neurons. Importantly, increased extracellular Hsp60 observed at 12 and 48 hours following MPP^+^ treatment was not due to permeabilization of cell membranes as the release of LDH (a determinant of cell membrane integrity) was only significant after 24 hours of treatment (Figure 2C). By contrast, decreased mitochondrial cell function evidenced via the MTT assay [37] was detected earlier (six hours) after MPP^+^ treatment and concomitant with the rise in extracellular Hsp60. Altogether, these results indicate that MPP^+^ treatment of SH-SY5Y cells compromised their function and stimulated the release of Hsp60 well before membrane rupture and death, suggesting the existence of an active Hsp60 secretory mechanism when DA neurons are in a suffering/damaged state.

### Hsp60-induced glial cell activation, mediates DA cell death and exacerbates MPP^+^ toxicity in mesencephalic cultures

**Figure 3:**
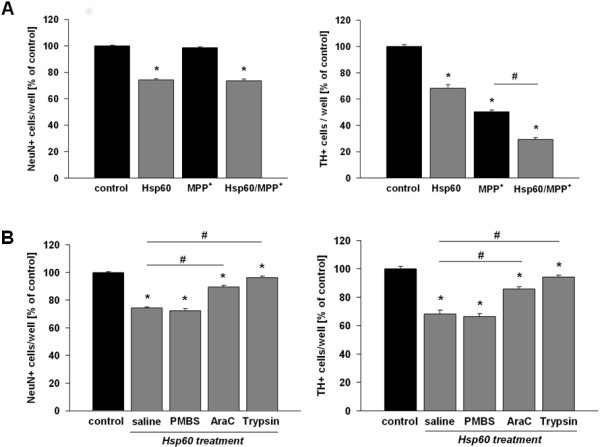
**Induction of dopaminergic (DA) cell death by Hsp60. (A)** Survival of TH^+^ neurons in mesencephalic neuron-glia cultures treated either with MPP^+^ (1 μM), hHsp60 (10 μg/ml) or a combination of both. **(B)** Survival of TH^+^ neurons in mesencephalic neuron-glia cultures exposed to native hHsp60 (10 μg/ml) alone or in the presence of thelipopolysaccharide antagonist PMBS (10 μg/ml), or to trypsinized hHsp60. hHsp60 was also applied to AraC (2 μM)-treated cultures, a treatment that eliminates most of glial cells from mesencephalic cultures. *P < 0.05 versus corresponding controls. #*P* < 0.05 versus hHsp60 treatment.

We next addressed the question whether Hsp60 influences neuronal cell death and treated primary mesencephalic cultures with either 1 μM MPP^+^ at DIV 4 and DIV 5, 10 μg/ml hHsp60 at DIV 2 to 5, or a combination of both. At DIV 5, medium including MPP^+^ and hHsp60 was removed and cultures were left to recover until DIV 10 in control medium. Under these experimental conditions, hHsp60 treatment decreased TH^+^ neurons to 68.3% ± 2.6% compared to untreated cultures and the overall population of neurons (NeuN^+^ cells) of these cultures to 74.4% ± 0.7% (Figure 3A). Treatment with MPP^+^ decreased the number of TH^+^ cells to 50.3% ± 1.4%, but did not affect the number of NeuN^+^ cells (98.6% ± 0.7%). Primary mesencephalic cultures contain only 1 to 3% of DA neurons (TH^+^) [38]. These neurons are selectively killed by MPP^+^ at the dosage used, leaving intact the remaining neuronal populations. Thus, the number of NeuN^+^ positive neurons was not significantly affected by MPP^+^ treatment.

Interestingly, co-treatment with hHsp60 and MPP^+^ led to a further loss of TH^+^ neurons in comparison to MPP^+^ or hHsp60 treatment alone (MPP^+^: 50.3% ± 1.4%, hHsp60: 68.3% ± 2.6 co-treatment of MPP^+^/hHsp60: 29.4% ± 1.3%), but exerted no additional effect on NeuN^+^ cells (co-treatment of MPP^+^/hHsp60: 73.6% ± 1.3%, MPP^+^: 98.6% ± 0.7%, hHsp60: 74.4% ± 0.7%) (Figure 3A).

To determine whether neuronal cell death mediated by Hsp60 required glial cell activation, we inhibited the proliferation of glial cells with the anti-mitotic agent AraC (2 μM) applied at DIV 1 for 48 hours [28]. The elimination of glial cells from the cultures curtailed neuronal cell death induced by Hsp60 (Figure 3B). Besides, hHsp60-dependent neurotoxic effects were almost abrogated when hHsp60 was previously denatured by trypsinization (TH^+^: 94.3% ± 1.2%), but not when cultures were exposed to both hHsp60 and the specific LPS antagonist PMBS (TH^+^: 66.3% ± 2.1%), suggesting that the effects of hHsp60 occurred by a mechanism unrelated to that elicited by LPS (Figure 3B).

### Hsp60 binds to microglia but does not induce pro-inflammatory cytokine release

**Figure 4:**
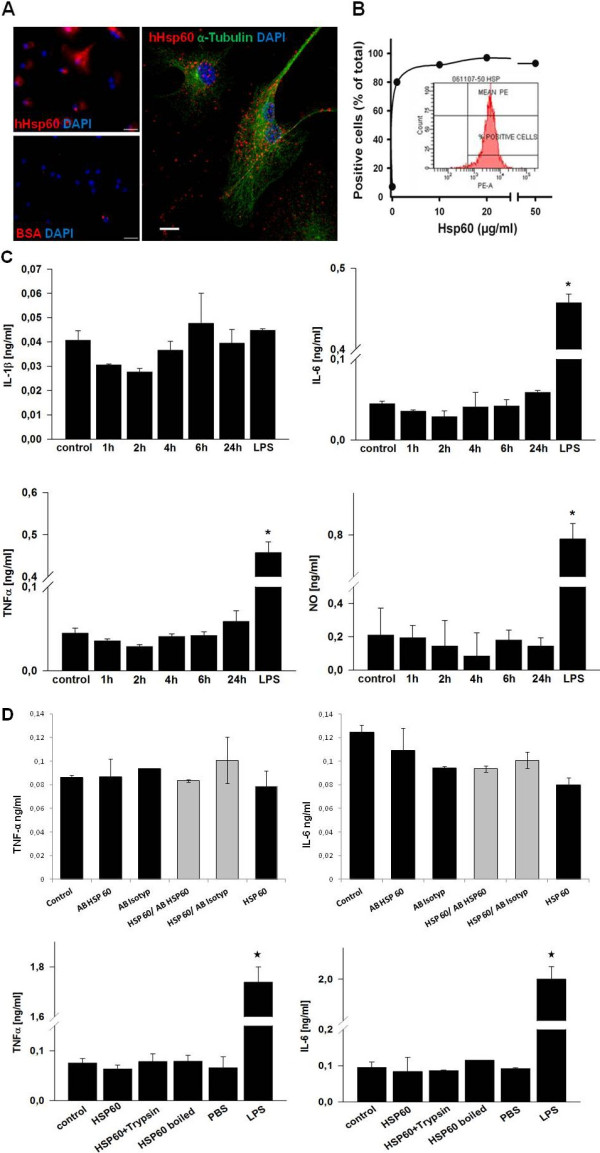
**Hsp60 and inflammatory processes mediated by microglial cells. (A)** Left: microglial cells exposed to Alexa647-conjugated hHsp60 (red label) or Alexa647-conjugated BSA (used as negative control) with 4’6-diamidino-2-phenylindole (DAPI) (blue label) counterstaining of cell nuclei. Scale bar = 50 μm. Right: confocal laser scanning microscopy of microglial cells that were exposed to Alexa647-conjugated Hsp60 (red label), then stained with an α-tubulin antibody (green label) and counterstained with DAPI (blue label). Scale bar = 10 μm. **(B)** Measure of cell surface binding of hHsp60 using extracellular FACS analysis of microglial cells labeled with an anti-Hsp60 monoclonal antibody (SPA-810, StressGen, San Diego, CA, USA) and a phycoerythrin-labeled secondary antibody. **(C)** Microglial cells were treated with hHsp60 at a concentration of 10 μg/ml, after 1, 2, 4, 6 and 24 hours respectively. Lipopolysaccharide (LPS) treatment for 6 hours for cytokine release respectively 24 hours for nitric oxide (NO)-release in a concentration of 0.1 μg/ml was used as positive control. **(D)** In addition, microglial cells were exposed to different hHsp60 preparations (native hHsp60, hHsp60 denatured by trypsinization or boiling and hHsp60 pre-incubated with Hsp60-specific antibody or isotype control antibody), after 12 and 24 hours, respectively. lipopolysaccharide (LPS) treatment for 12 hours in a concentration of 0.1 μg/ml was used as positive control, PBS as buffer control.

A direct biological effect of Hsp60 on microglia requires the binding to specific receptors. Therefore, to determine whether Hsp60 could provoke microglial cell-activating properties similar to those previously reported for macrophages [39,40], we performed *in vitro* stimulation of primary microglial cell cultures with recombinant hHsp60. For comparative purposes, control cultures were exposed to LPS. Since Hsp60 is thought to induce a pro-inflammatory response through binding to the LPS receptor TLR4 [41], we first analyzed the binding properties of hHsp60 on murine microglia. Incubation of microglial cells with Alexa647-conjugated hHsp60, but not with Alexa647-conjugated BSA used as a negative control, revealed a punctuated distribution of bound hHsp60 suggesting the presence of cell surface receptors that binds to the protein (Figure 4A). Fluorescence-activated cell sorting (FACS) analysis of cells incubated with increasing concentrations of hHsp60 indicated a non linear and saturable binding of hHsp60 to microglia, thus further supporting the view that microglial cells express specific Hsp60 receptors (Figure 4B).

Subsequently, microglial cells were treated with hHsp60 at a concentration of 10 μg/ml. After 1, 2, 4, 6 and 24 hours respectively, hHsp60-exposed cells showed no morphological alterations (that is enlargement of cell bodies; data not shown). Furthermore, they did not release significantly increased amounts of pro-inflammatory cytokines (IL-6, IL-1β and TNF-α) or NO compared to the control condition (Figure 4C). In the LPS-treated positive control (0.1 μg/ml), significant values of IL-6 and TNF-α could be shown after 6 and 24 hours respectively for NO. However, a significant increase of IL-1β could not be shown in the LPS-treated group. This may be due to concentrations of LPS used being too low and/or the time of measurement being too early (six hours) to sufficiently stimulate IL-1β release.

In addition, microglial cells were exposed to different hHsp60 preparations (native hHsp60, hHsp60 denatured by trypsinization or boiling and hHsp60 pre-incubated with Hsp60-specific antibody or isotype control antibody). After 12 and 24 hours respectively, hHsp60-exposed cells did not release significantly increased amounts of pro-inflammatory cytokines (IL-6 and TNF-α) compared to the control condition. (Figure 4D). Taken together, these observations strongly suggest that microglia express cell surface receptors to interact with Hsp60. Microglia, however, do not react to Hsp60 with the production of measurable amounts of classical pro-inflammatory cytokines such as IL-1β, IL-6, TNF-α or NO release.

## Discussion

Experimental data presented here suggest that Hsp60 might participate actively in DA cell demise in PD. First, *in vivo*, we observed that Hsp60-mRNA was up-regulated in the mouse mesencephalon and striatum in a toxin-based model of PD, as well as in the SN of PD patients. Second, *in vitro*, we showed that MPP^+^ increases the expression and release of Hsp60 from DA cells and exacerbates MPP^+^-induced DA cell death. Surprisingly, however, despite this specific binding to microglia already been shown by Lehnardt *et al*. [19], hHsp60 did not cause induction of pro-inflammatory cytokines.

Hsps were initially discovered as participants in the cellular response to stress [42]. Other studies suggested that self and microbial Hsps may also play an important role in the control of the immune response [43]. In particular, Hsps including Hsp60, Hsp70 and gp96 released by injured or dying cells, are believed to function as endogenous danger signals to the immune system indicating tissue injury [44-47]. Here, we evaluated the potential role of Hsp60 in DA cell demise in PD, a pathological condition in which inflammatory processes are thought to contribute actively to degeneration.

Using the acute MPTP mouse model, characterized by a robust gliosis in the SNpc with significant up-regulation of inducible NOS and activation of microglia [48], we demonstrated that Hsp60 mRNA expression is up-regulated in the mesencephalon and the striatum after MPTP intoxication, suggesting that Hsp60 had the potential to operate as an endogenous activator of microglia in animal models of PD. Supporting this view, the kinetics of Hsp60 up-regulation after MPTP intoxication resembled that of CD11b/Mac1 induction which reflects microglial activation [48]. Interestingly, we found that in the SN of PD patients, Hsp60 mRNA also up-regulated compared to control subjects indicating that the observations made with the MPTP mouse model might be of relevance for the human pathology.

The assumption that Hsp60 could intervene actively in the progression of the disease was also supported by several observations made in two complementary cell culture settings; (i) we found that mitochondrial stress triggered by MPP^+^ caused an up-regulation and release of Hsp60 in human DA SH-SY5Y cells well before the onset of neuronal cell demise; in our model system, the release of Hsp60 was already detectable in the supernatant of cells with intact plasma membranes, so that cell lysis would not be necessary for the accumulation of Hsp60 in the extracellular milieu, (ii) application of Hsp60 to primary mesencephalic cultures triggered DA cell death *per se* and importantly, it also exacerbated the toxicity of MPP^+^ for these neurons.

The stimulation of TLR4 by LPS induces the release of critical pro-inflammatory cytokines that are necessary to activate potent immune responses. Recently, we could demonstrate that TLR4-deficient mice are less vulnerable to MPTP intoxication than wild-type mice and display a decreased number of activated microglial cells after MPTP application, suggesting that the TLR4 pathway is involved in experimental PD. However, the endogenous activator of TLR4 in this scenario could not be identified [49]. A number of arguments suggested that Hsp60 could be a potential ligand for TLR4, which is expressed on microglia, astrocytes and endothelial cells in the central nervous system [50-53]. In particular, extracellular eukaryotic Hsp60 was found to induce the release of pro-inflammatory cytokines such as IL-1, IL-6, IL-12, and TNF-α in dendritic cells and macrophages [43,45,54,55]. Lehnardt *et al*. determined in a previous study that the neurotoxic effect of Hsp60 requires the presence of microglia. Additionally, Lehnardt *et al*. could show that microglia bind Hsp60-Alexa, whereas neurons, oligodendrocytes and astrocytes did not show any labeling with Hsp60-Alexa [19]. Therefore, we have placed the focus in our study on microglia and not on astrocytes or endothelial cells.

Here, we observed an induction of Hsp60 after MPTP intoxication. Furthermore, we established that Hsp60 binds specifically to microglia in a saturable-dependent manner, suggesting that the protein could operate as an endogenous activator of TLR4, thus stimulating inflammatory processes. In contrast to the latter, Hsp60 did not induce the release of detectable amounts of cytokines. However, low levels of pro-inflammatory cytokines that are below detection limit may be induced by Hsp60 and might well exert biological activity in a certain microenvironment: that is the direct contact of microglia with neighbouring neuronal cells. In addition, Hsp60 may induce the production of stimulatory or toxic molecules other than IL-1β, IL-6, TNF-α or NO that were not analyzed in this study.

Taking into consideration that Hsp60 activates murine antigen-presenting cells (APC) also via TLR4 independent pathways in the absence of LPS or other so called pathogen-associated molecular patterns (PAMPs) [41,56], it is also possible that the Hsp60 acts via other receptors such as the lectin-like oxidized low-density lipoprotein receptor-1 (LOX-1) previously identified as a putative receptor for Hsp60 in microglial cells [57]. Other PAMP could be involved in the stimulation of the innate immune system in some types of sterile inflammation, like PD, which need to be explored in further studies.

Alternatively, a direct effect of Hsp60 on DA neurons remains also conceivable. Yet, the requirement of glial cells in the effects of Hsp60 appeared crucial as its toxic effects towards DA neurons were curtailed in neuronal-enriched cultures.

## Conclusions

Taken together, we could demonstrate a deleterious role of Hsp60 on DA neurons in different experimental PD models. However, our data do not support that Hsp60 is an endogenous activator of the TLR4-pathway in experimental PD models and, by extrapolation, in the human disease. Thus the cellular target of Hsp60 remains to be identified.

## Abbreviations

ANOVA: analysis of variance; APC: antigen-presenting cells; Ara-C: cytosine arabinoside; BSA: bovine serum albumin; CD11b: cluster of differentiation molecule 11B; CNS: central nervous system; DA: dopaminergic; DAPI: 4’6-diamidino-2-phenylindole; DIV: days *in vitro*; DMEM: Dulbecco's Modified Eagle's Medium; DNA: deoxyribonucleic acid; ECM: major extracellular matrix; ELISA: Enzyme Linked Immunosorbent Assay; FACS: Fluorescence-activated cell sorting; FCS: fetal calf serum; GAPDH: Glyceraldehyde 3-phosphate dehydrogenase; hHsp60: human heat shock protein 60; HPRT: Hypoxanthine-guanine phosphoribosyltransferase; HRP: Horseradish peroxidase; Hsp60: heat shock protein 60; Hsps: heat shock proteins; IL-1β: interleukin-1 beta; IL-6: interleukin-6; LDH: lactate dehydrogenase; LPS: lipopolysaccharide; Mac1: Macrophage-1 antigen; MK-801: Dizocilpine; MPP+: 1-methyl-4-phenylpyridinium; MPTP: 1-methyl-4-phenyl-1,2,3,6-tetrahydropyridine; MTT: 3-[4,5-dimethylthiazol-2-yl]-2,5-diphenyl-tetrazolium bromide; MyD88: myeloid differentiation factor 88; NeuN: neuronal Nuclei; NO: nitric oxide; PAMPs: pathogen-associated molecular patterns; PBS: phosphate buffered saline; PD: Parkinson disease; PEI: polyethylenimine; PFA: paraformaldehyde; PMBS: polymyxin B; RNA: ribonucleic acid; SH-SY5Y: human neuroblastoma cell line; SN: substantia nigra; SNpc: substantia nigra pars compacta; TH: tyrosine hydroxylase; TLR4: Toll-like receptor 4; TLRs: Toll-like receptors; TNF-α: tumor necrosis factor-alpha.

## Competing interests

The authors declare that they have no competing interests.

## Authors’ contributions

The work presented here was carried out in collaboration between all authors. CN, SH and AH defined the research theme. CN, SH and AH designed methods and experiments, carried out the laboratory experiments, analyzed the data, interpreted the results and wrote the paper. LM, DAF, TL, CH and LL co-designed experiments, discussed analyses, interpretation, and presentation. RCD and ECH analyzed the data and interpreted the results. AO, MB and PPM co-worked on associated data collection and their interpretation. All authors have contributed, seen and approved the manuscript.
